# A Genome Wide Association Study of Mathematical Ability Reveals an Association at Chromosome 3q29, a Locus Associated with Autism and Learning Difficulties: A Preliminary Study

**DOI:** 10.1371/journal.pone.0096374

**Published:** 2014-05-06

**Authors:** Simon Baron-Cohen, Laura Murphy, Bhismadev Chakrabarti, Ian Craig, Uma Mallya, Silvia Lakatošová, Karola Rehnstrom, Leena Peltonen, Sally Wheelwright, Carrie Allison, Simon E. Fisher, Varun Warrier

**Affiliations:** 1 Autism Research Centre, Department of Psychiatry, University of Cambridge, Cambridgeshire, United Kingdom; 2 CLASS Clinic, Cambridgeshire and Peterborough NHS Foundation Trust (CPFT), Cambridgeshire, United Kingdom; 3 School of Psychology and Clinical Language Sciences, Centre for Integrative Neuroscience and Neurodynamics, University of Reading, Reading, United Kingdom; 4 MRC Centre for Social, Genetic and Developmental Psychiatry, King's College London, Institute of Psychiatry, London, United Kingdom; 5 The Wellcome Trust Sanger Institute, Hinxton, Cambridgeshire, United Kingdom; 6 Max Planck Institute for Psycholinguistics, 6500 AH Nijmegen, The Netherlands; 7 Donders Institute for Brain, Cognition and Behaviour, Radboud University Nijmegen, Nijmegen, The Netherlands; University of Texas School of Public Health, United States of America

## Abstract

Mathematical ability is heritable, but few studies have directly investigated its molecular genetic basis. Here we aimed to identify specific genetic contributions to variation in mathematical ability. We carried out a genome wide association scan using pooled DNA in two groups of U.K. samples, based on end of secondary/high school national academic exam achievement: high (n = 419) versus low (n = 183) mathematical ability while controlling for their verbal ability. Significant differences in allele frequencies between these groups were searched for in 906,600 SNPs using the Affymetrix GeneChip Human Mapping version 6.0 array. After meeting a threshold of p<1.5×10^−5^, 12 SNPs from the pooled association analysis were individually genotyped in 542 of the participants and analyzed to validate the initial associations (lowest p-value 1.14 ×10^−6^). In this analysis, one of the SNPs (rs789859) showed significant association after Bonferroni correction, and four (rs10873824, rs4144887, rs12130910 rs2809115) were nominally significant (lowest p-value 3.278 × 10^−4^). Three of the SNPs of interest are located within, or near to, known genes (*FAM43A*, *SFT2D1*, *C14orf64*). The SNP that showed the strongest association, rs789859, is located in a region on chromosome 3q29 that has been previously linked to learning difficulties and autism. rs789859 lies 1.3 kbp downstream of *LSG1*, and 700 bp upstream of *FAM43A*, mapping within the potential promoter/regulatory region of the latter. To our knowledge, this is only the second study to investigate the association of genetic variants with mathematical ability, and it highlights a number of interesting markers for future study.

## Introduction

Mathematics is the basis of science, technology, engineering, and at complex levels (e.g. number theory, algebra) is uniquely human. Despite its obvious importance, our understanding of what gives rise to individual differences in mathematical ability has not been widely studied. Mathematical talent clusters in families [1] and heritability studies indicate that this is in part due to genetic factors. A wide range of estimates have been reported for the proportion of variation in mathematical ability accounted for by genetic factors, from 0.2 up to 0.9 [2]–[5]. The large variance may be due to different phenotypic measures used, as mathematics is not unitary, so different phenotypic measures may tap distinct components. Academic achievement [5], standardized test scores [2] and teacher reported skills [4] have all been used to measure aptitude for mathematics in quantitative genetic studies.

To our knowledge, only one genetic association study with mathematical ability has been published thus far. DNA pooling was used in two separate sample sets to test for statistically significant differences in allele frequency using a microarray with 500 K SNPs. The comparison was between participants classified as having high or low mathematical ability (measured using a web-based test performance as well as teacher ratings) who were 10 years old at time of testing. The 43 SNPs that most highly differed in frequency between the phenotype groups in the pools were chosen for individual genotyping using a sample set representing the distribution of mathematical ability as a quantitative trait. Ten of these 43 were found to be nominally associated (p-value<0.05) [6].

In the present study we extended the search for genetic factors for mathematical ability by using pooled DNA from participants who all had excellent verbal ability but who differed in terms of having either high or low mathematical ability, to look for association with any of 906,600 SNPs across the genome. There were four key differences from the Docherty et al. (2010) study: 1) We controlled for verbal ability while taking high and low ends of mathematical ability; 2) the phenotypic measure was based on standardized school examination performance; 3) the number of SNPs in the initial exploratory phase was nearly double that of the previous study; and 4) the mean age of the participants was significantly higher than that of Docherty et al. (2010). Twelve SNPs exceeded our designated threshold for significance (p-value<1.5×10^−5^) at the pooling stage and were individually genotyped and analyzed in the sample to validate the associations. This step also allowed us to evaluate the efficacy of the DNA pooling method in predicting allele frequency.

## Methods

### Ethics Statement

Ethical approval was obtained from the University of Cambridge Psychology Research Ethics Committee, and all participants provided written informed written consent. All participants in this study were above 16 years of age and written informed consent was obtained from the participant.

### Participants

602 participants from the U.K. were recruited by advertisement from local sixth form schools (n = 230) and universities (n = 372) between 2004 and 2008. Participants were included in the study if they reported Caucasian ancestry for 2 generations and had no psychiatric and/or neurological conditions. All individuals were at least 16 years old.

Each individual was classed as having high or low mathematical ability depending on their General Certificate Standard Examination (GCSE) results for Mathematics. To be included in the study the Mathematics GCSE result for the high maths group had to be an A or A* grade (n  =  419, 216 males and 203 females). To be included in the low maths group the Mathematics GCSE result had to be a C grade or below (n  =  183, 50 males and 133 females). For both of these ability groups, the person's GCSE English grade had to be an A or A*. This was included to control for verbal ability and to ensure we were not just testing for genetic associations with general academic aptitude. The high maths group included some university students, studying a ‘hard’ science or mathematical degree and had also obtained an A grade at GCE Advanced Level (A-level). The low maths group also included some university students but they were studying a Humanities degree courses had not taken mathematics at A- level or had received a C or below grade. In this way the two maths groups were matched for both age and proportion in university education. Verbal ability for all individuals was high as they all had an A grade or above in English.

### Pooling stage

Genomic DNA from each individual was extracted from buccal swabs supplied by the individuals and then anonymized. The DNA was then suspended in Tris-ethylenediamineaacetic acid (EDTA) (TE) buffer (0.01 M tris-hCl, 0.001 M EDTA, pH 8.0) and quantified using PicoGreen double-stranded DNA quantification reagent (Invitrogen, USA). An equimolar amount of DNA (100 ng) from each individual was added to his or her respective pool. There were 10 high mathematical ability pools (5 female only and 5 male only) and 5 low mathematical ability pools (3 female only and 2 male only) with a mean of 40.1 (SD =  12.1) individuals per pool. These pools were interrogated using an Affymetrix GeneChip Human Mapping version 6.0 array (Affymetrix, California, USA) using the standard Affymetrix protocol. Washing and staining was performed using the Fluidics Station 450 and scanned using the GeneChip Scanner 3000 7G, which was controlled using GENCHIP operating software (GCOS) generating cell intensity (.cel) files. The files generated were converted into relative allele signal (RAS) scores using a custom-made statistical protocol in R, snpmap.R [7].

Independent t-tests on the mean RAS scores from the pools for each SNP were performed between the different groups (high mathematical ability and low mathematical ability) to test for significant allele frequency differences. To be chosen for validation by individual genotyping, the difference between allele frequencies for any one SNP was required to have a p-value below 1.5×10^−5^. This is a relatively lenient p-value threshold in the context of a genome-wide screen, but it was chosen *a priori* in order to reduce the risk of false negatives. Due to the low power retained in DNA pooling of the sample [8] causative SNPs are likely to be missed when adopting a higher threshold for taking them forward to validation. SNPs were rejected for the individual genotyping stage if their minor allele frequency (MAF) in the Caucasian population was below 0.01 as the study did not have the power to pick up rare variants. Post-hoc power calculation was performed using genetic power calculator [9]. Power was calculated using case-control for threshold selected quantitative traits option. QTL variance explained by each SNP was assumed to be 0.01. The frequencies of the increaser allele and the marker allele was assumed at 0.2 each, and the LD (D′) between the marker and the increaser allele was 0.8. At *P* < 0.05, the power at this stage was 61% under an additive model. SNPs were also rejected depending on the calculated coefficient of variation (CV) of the RAS scores for the SNP. If 50% of the pools showed CV >20 for the SNP then it was rejected from analysis.

### Individual genotyping stage

All genotyping was carried out by Geneservices UK Ltd using the Sequenom MassARRAY iPLEX platform (Sequenom, San Diego, USA). Out of the 592 individuals screened in the pooling stage, 542 were available for the individual genotyping stage (high maths group n  =  375, 194 males, 181 females; low maths group n  =  167, 40 males, 127 females). 60 individuals were not individually genotyped due to the lack of DNA. To check if the missing individuals significantly altered the composition of the groups between the pooling and the individual genotyping stage, a chi-square test was performed and the results were non-significant at three degrees of freedom (chi-sq  =  6.89; P-value  =  0.07). Four individuals were excluded for having over 10% genotyping data missing in the individual genotyping stage and two individuals were excluded due to uncertainty over phenotype status. However, since they were present in different pools during the pooling stage, it is unlikely that they greatly influenced the results of the pooling stage. Out of the 15 SNPs that were selected for individual genotyping, three SNPs had <90% genotyping success in this stage of work, and hence were excluded (noted in Table 1). For the remaining 12 SNPs, no marker deviated significantly from Hardy-Weinberg equilibrium (p<0.001).

**Table 1:** 15 SNPs chosen for individual genotyping with the P-values from the pooling stage.

rsID	Chr	Nearest gene	Pooling P-value	Chromosome Position	Allele1	Allele2	Allele 1 Frequency	Allele 2 Frequency
rs12130910	1	*RP11-815M8.1*	5.04× 10^−06^	222038372	G	A	0.647	0.353
rs11808800	1	*NTNG1*	9.41×10^−06^	108040994	T	C	0.973	0.027
rs10873824	1	*HS2ST1*	1.42×10^−05^	87688394	A	G	0.283	0.717
rs10179572^a^	2	*C2orf83*	1.31×10^−05^	228504503	A	G	0.535	0.465
rs6546878	2	*DGUOK*	1.02×10^−05^	74175324	C	A	0.248	0.752
rs12629229	3	*FRMD4B*	1.07×10^−05^	69411222	T	C	0.765	0.235
rs789859	3	*FAM43A*	4.57×10^−06^	194405888	G	T	0.611	0.389
rs9884781^a^	4	*AC007106.1*	9.80×10^−06^	27989923	T	A	0.535	0.465
rs13355548	5	*RP11-152K4.2*	1.14×10^−06^	31089349	G	A	0.875	0.125
rs2974097	5	*CTD-2036A18.2*	1.30×10^−05^	85333953	T	A	0.58	0.42
rs9400442^a^	6	*RPF2*	3.42×10^−06^	111298225	A	G	0.823	0.177
rs4144887	6	*SFT2D1*	3.19×10^−06^	166747512	G	A	0.748	0.252
rs973582	8	*SGCZ*	1.50×10^−05^	13909182	G	A	0.69	0.31
rs2138861	12	*RP11-25I15.3*	3.96×10^−06^	43166458	A	T	0.704	0.296
rs2809115	14	*C14orf64*	6.89×10^−06^	98406425	A	T	0.559	0.441

a Indicates SNPs that did not survive quality control measures to be included in the association validation analysis.

### Validation of association

To validate the results of association found in the pooling stage, association analysis was carried out on the individual genotype data using PLINK version 1.07 [10]. For each of the 12 SNPs that were successfully genotyped, a Cochran-Armitage trend test (1df) was performed, under the null hypothesis that the allele frequencies did not deviate significantly between the different ability groups. A Bonferroni correction was applied to correct for multiple testing of 12 different SNPs in this stage. Thus, we considered a result to be significant in this validation analysis if it yielded a p-value below 0.0042 (0.05/12).

## Results

**Figure 1:**
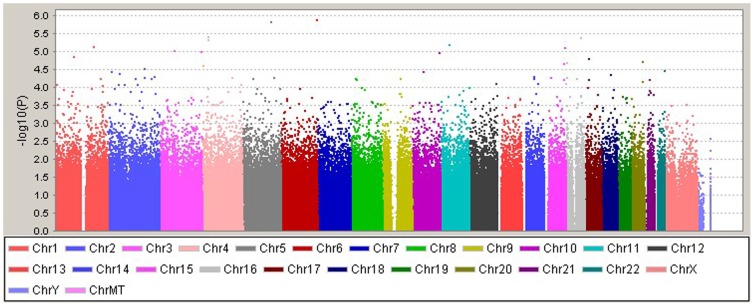
Graphical summary of association results from the genome-wide screen of pooled samples. X-axis represents the chromosome position; y-axis shows -log10 of the P-value obtained for each SNP.

Analysis of the mean RAS scores from the DNA pools identified 15 SNPs with evidence of association that passed our pre-designated significance threshold of p<1.5×10^−5^ (see Figure 1). All 15 SNPs were taken forward to the next stage of individual genotyping (see Table 1).

**Figure 2:**
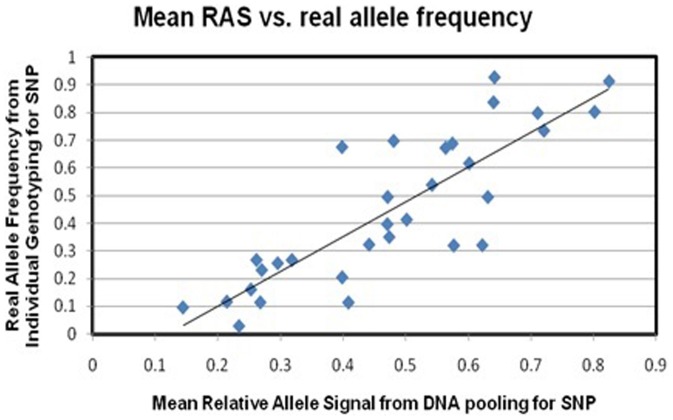
Pearson's correlation of real allele frequencies in total sample (calculated from individual genotyping,y-axis) and frequency estimates from pooled DNA (mean RAS-scores of the 15 pools, x-axis) of 32 SNPs (r  =  0.8424).

To assess the ability of DNA pooling to accurately predict the allele frequency in these individuals, the real allele frequencies of 31 SNPs (the 12 SNPs that met our significance threshold as well as 19 other SNPs) individually genotyped were compared with the mean RAS scores calculated for these SNPs from the DNA pooling stage. Pearson's correlation coefficient for the correlation was r  =  0.8424 (see Figure 2). The 31 SNPs and the rationale for choosing them is given in File S1.

Of the 15 SNPs chosen for individual genotyping, 12 survived quality control at the individual genotyping stage and were analyzed. Five SNPs were nominally significant, with one of them remaining significant after correcting for multiple comparisons of 12 SNPs in this stage (see Table 2). Three of these SNPs map near to, or within, known genes (*FAM43A*, *SFT2D1*, *C14orf64*). The most significant SNP in our study, rs789859 (p-value: 0.000328, Odds ratio: 1.629), is located 700 bp upstream of *FAM43A*.

**Table 2:** Association results of SNPs associated at a significance value of 0.05 or less in the individual genotyping stage, along with their pooling stage results.

Variation	Nearest Gene	DNA Pooling		Individual Genotyping	
		P-value	X2	P-value (additive)	Odds ratios (with 95% confidence intervals)
rs789859	*FAM43A*	4.57×10^−6^	12.9	0.000328	1.629 (2.129–1.247)
rs4144887	*SFT2D1*	3.19 ×10^−6^	6.66	0.009838	1.488 (2.014–1.099)
rs12130910	*RP11-815M8.1*	5.04 ×10^−6^	4.57	0.03242	1.353 (1.785–1.044)
rs2809115	*C14orf64*	6.89 ×10^−6^	4.27	0.03878	1.318 (1.714–1.014)
rs10873824	*HS2ST1*	1.42 ×10^−5^	3.89	0.04848	1.343 (1.801–1.002)

SNPs written in bold are significant after correcting for multiple testing.

## Discussion

The present study involved a genome-wide screen for association with mathematical ability in a general population sample. In a pooling based genome-wide screen, 15 SNPs were associated with a p < 1.5 × 10^−5^, leading to follow up individual genotyping and analysis of 12 of these SNPS. This revealed five SNPs to be nominally significant (p < 0.05), one of which remained significant after Bonferroni correction for testing multiple markers in the individual genotyping stage. Three of these SNPs are located close to known genes (*FAM43A*, *SFT2D1*, *C14orf64*).

The SNP with the highest significance, rs789859, can have a G or T allele and is located in an intergenic region on chromosome 3q29. Microdeletions and duplications in this region have been associated with autism, schizophrenia as well as learning difficulties [11]–[14]. rs789859 is approximately 700 bp upstream of *FAM43A*,within its 5′ –regulatory region, and 1.3 kbp downstream of *LSG1*. The fact that rs789859 is located in a region that has been previously associated with neurological conditions affecting associative learning [12] is consistent with a putative contribution to mathematical ability. *FAM43A* is a plausible candidate gene, since the SNP maps within the potential promoter/regulatory region of this gene. rs789859 is in high LD with six other variants when queried in Haploreg. Two of these variants (rs150293579 and rs1675923) map within *LSG1*. Both these variants regulate chromatin states in various cell types from the human CNS. The remaining four variants map near *FAM43A* and also regulate chromatin states in brain cell types and alter TF binding sites. The web resource FASTSNP [15] predicts this SNP to be in a possible transcription-factor binding region. *FAM43A* has thus far only been characterized in cDNA assays and is predicted to encode a hypothetical protein, LOC131583, with little knowledge about its function. RNA expression assays have found *FAM43A* RNA in a variety of tissues including tissues from the brain, cerebellum and spinal cord. Since *FAM43A* is not a well-characterized gene at this stage, conclusions about its viability as a candidate, and its possible contributions to the phenotype are not easily reached.

There was no overlap between the SNPs associated in this study and those reported in the only previously published molecular screen of mathematics abilities. This is not surprising given that such abilities must have a complex genetic basis with multiple genetic factors of small effect size, and both studies involved relatively small sample sizes (further discussed below). Methodological differences between the studies may also have contributed to the lack of overlap. The phenotype in this investigation was standardized national exam performance at age 16–18 years old, where the previous published association study [6] employed a composite score based on web-based testing and teacher report of 10-year-olds. Heritability studies of mathematical ability and how it changes with age may indicate whether age should be an important issue in experimental design for future association _studies._

A limitation of this study is its statistical power. The sample size is small to robustly detect causative loci for a complex trait. Due to the expected small effect sizes of the variants underlying mathematics ability, and complexity of the phenotype tested, larger samples will be needed to definitively identify causative variants. In addition, a previous study using a pooling method suggested the power retained is 68% of the sample [8]. Considering the relatively high correlation of the RAS scores from DNA pooling to real allele frequency demonstrated in this study, the failure of a majority of the SNPs chosen for individual genotyping to reach expected thresholds of significance reflects this lack of power. Quality control exclusions and the fact that a number of individuals from the pooled analysis were not available for individual genotyping are other issues affecting this. The correlation to compare pooling estimates to real allele frequencies was conducted by calculating means of the RAS scores for each SNP from all the pools and thus all the individuals together, so that the missing individuals would have a lesser effect on the numbers than if the correlations were conducted between the phenotype groups separately. These issues may have contributed to the weaker evidence for association observed in the validation step compared to the p-values seen in the DNA pooling GWA study.

In addition to pointing to genetic variation potentially linked to mathematical ability, this study also provides methodological insights for genetic association studies that use pooled DNA. To our knowledge, there is one other published DNA pooling study which used the same Affymetrix GeneChip Human Mapping version 6.0 array [16]. In contrast to the results obtained in this study, which reported a low correlation between allele frequency estimated by the pooled DNA analysis and the real allele frequency determined by individual genotyping (Pearson's r =  0.2734), we report a higher correlation of 0.8424, using the same technique (using DNA from buccal swabs). This improved correlation between estimated and real allele frequencies in this study could be due to a) higher DNA quality in this study, and b) the fact that 110 SNPs were individual genotyped by Schosser et al. 2010, while the current paper only included 32 SNPs. However, the higher correlation in the current analysis for the subset of 32 SNPs suggests that the inferences drawn about these 32 SNPs are reliable. Thus, is it suggested that this microarray may be more suitable for using a DNA pooling approach with buccal mucosa swab samples than previously reported.

In conclusion, in this study we detect new candidate loci that might be associated with mathematical ability. The SNP showing the strongest association is located in a genetic region (3q29) that has been proposed to be associated with autism, schizophrenia and learning difficulties [12]–[14]. Potential future studies could explore the phenotypic overlap (or non-overlap) between mathematical ability and different psychopathological conditions. The known association between autism spectrum conditions and mathematical ability [17], [18] suggests that these SNPs should also be tested for association with autism. We acknowledge that the sample size is small for robustly detecting loci with small effect sizes, and research in larger, independent samples should be conducted to further delineate the genetic architecture that contributes to mathematical ability in the general population.

## Supporting Information

File S1 **This file contains Table S1 and Table S2.** Table S1 provides the results of the sex-stratified analyses for the pooling stage. Table S2 provides the list of SNPs used to assess the accuracy of the pooling stage.
(DOCX)
