# Supplementary material for: A Genome Wide Association Study of Mathematical Ability Reveals an Association at Chromosome 3q29, a Locus Associated with Autism and Learning Difficulties: A Preliminary Study
Source: PLoS One. 2014 May 6;9(5):e96374. doi: 10.1371/journal.pone.0096374 (PMC4011843; doi:10.1371/journal.pone.0096374)
Supplement: File S1 — This file contains Table S1 and Table S2. Table S1 provides the results of the sex-stratified analyses for the pooling stage. Table S2 provides the list of SNPs used to assess the accuracy of the pooling stage. (DOCX) [file pone.0096374.s001.docx]

Supporting information

**A genome wide association study of mathematical ability reveals an association at chromosome 3q29, a locus associated with autism and learning difficulties: a preliminary study**

The additional 19 additional SNPs chosen to assess the accuracy of DNA were selected from the top performing SNPs from sex-stratified analysis. Due to the very small sample size, we choose SNPs that were below a P value of 1.5x10^-05^, and if multiple SNPs from the same gene had a P-value of below 1.5x 10^-4^. The SNPs and the P-values are given in table 1.

Table S1: List of SNPs that were significant at the pooling stage for sex-stratified analyses.

| SNP | Chromosome | P-value (pooled) |
| --- | --- | --- |
| Males only |  |  |
| rs12729364 | 1 | 1.53x10-05 |
| rs17019407 | 3 | 7.34x10-06 |
| rs1374837 | 3 | 4.57x10-05 |
| rs7626112 | 3 | 1.04x10-04 |
| rs9312642 | 4 | 3.73x10-06 |
| rs6838666 | 4 | 3.68x10-05 |
| rs10516081 | 5 | 7.8x10-06 |
| rs10520084 | 5 | 1.38x10-05 |
| rs9354097 | 6 | 1.44x10-06 |
| rs2402455 | 7 | 6.9x10-06 |
| rs10091890 | 8 | 1.31x10-06 |
| rs11790027 | 9 | 1.13x10-05 |
| rs12571782 | 10 | 1.27x10-05 |
| rs7924025 | 10 | 9.56x10-06 |
| rs7108801 | 11 | 6.51x10-06 |
| rs9534455 | 13 | 1.88x10-06 |
| rs12951898 | 17 | 9.53x10-07 |
| rs16984006 | 19 | 2.56x10-05 |
| rs10401904 | 19 | 1.46x10-04 |
| rs2827987 | 21 | 2.04x10-06 |
| rs738246 | 22 | 3.91x10-06 |
|  |  |  |
| Females only |  |  |
| rs10111041 | 8 | 1.47x10-04 |
| rs1559900 | 8 | 1.3x10-04 |

All the All SNPs were individually genotyped. Of these, all SNPs except rs1559900 (females), and rs9354097, rs7108801, and rs2827987 (males) failed quality control at the individual genotyping stage. No SNP passed Bonferroni correction at the individual genotyping stage (results not shown). The small sample size of the sex-stratified studies indicate that these analyses would not be sufficiently powered to identify variants with even moderate effect sizes.

All the SNPs that passed quality control in the sex stratified analyses and the combined analyses were used to assess the accuracy of the pooling stage. The final list of SNPs is given in Table 2.

Table S2: List of SNPs used to assess accuracy of pooling stage

| rsID | Analysis |
| --- | --- |
| rs12130910 | Combined |
| rs11808800 | Combined |
| rs10873824 | Combined |
| rs6546878 | Combined |
| rs12629229 | Combined |
| rs789859 | Combined |
| rs13355548 | Combined |
| rs2974097 | Combined |
| rs4144887 | Combined |
| rs973582 | Combined |
| rs2138861 | Combined |
| rs2809115 | Combined |
| rs12729364 | Males only |
| rs17019407 | Males only |
| rs1374837 | Males only |
| rs7626112 | Males only |
| rs9312642 | Males only |
| rs6838666 | Males only |
| rs10516081 | Males only |
| rs10520084 | Males only |
| rs2402455 | Males only |
| rs10091890 | Males only |
| rs11790027 | Males only |
| rs12571782 | Males only |
| rs7924025 | Males only |
| rs9534455 | Males only |
| rs12951898 | Males only |
| rs16984006 | Males only |
| rs10401904 | Males only |
| rs738246 | Males only |
| rs10111041 | Females only |
